# Secular Trend in the Physical Fitness of Xinjiang Children and Adolescents between 1985 and 2014

**DOI:** 10.3390/ijerph17072195

**Published:** 2020-03-25

**Authors:** Cunjian Bi, Feng Zhang, Yang Gu, Yi Song, Xiaodi Cai

**Affiliations:** 1 School of Physical Education and Health, East China Normal University, Shanghai 200241, China; cjbi1985@126.com; 2 Institute of Physical Education, Xinjiang Normal University, Wulumuqi 830054, China; xiangguyang09@sina.com (Y.G.); clxx0991@163.com (X.C.); 3 Institute of Child and Adolescent Health, School of Public Health, Peking University, Beijing 100191, China; songyi@bjmu.edu.cn

**Keywords:** physical fitness, nutrition status, Xinjiang, children and adolescents, physical fitness indicator, body mass index, growth status

## Abstract

We assessed trends in physical fitness by age group and nutrition status among children and adolescents in Xinjiang during 1985–2014. The data of 49,357 participants aged 7–18 were extracted in 1985, 1995, 2005, and 2014. Growth and nutritional status were defined using World Health Organization definitions. A physical fitness indicator (PFI) was calculated as the sum of six components measured in every survey. The relationship between body mass index (BMI) and PFI was investigated using nonlinear regression. Taking 1985 as a reference, PFI increased to 2 in 1995 and then fell sharply to −2.8 in 2005 and −3.8 in 2014. The prevalence of normal weight increased from 87.5% in 1985 to 89.4% in 1995 and then decreased to 75.2%, consistent with the change in PFI. The relationship between BMI and PFI showed an inverted U-shaped curve. The largest increment occurred in boys aged 13–15 and girls aged 16–18 during 1985–1995; the largest decline occurred in boys and girls aged 10–12 during 1995–2005. Our research provides a reference for local governments providing nutrition subsidies and projects in southern Xinjiang, indicating that greater attention is needed for children aged 7–12.

## 1. Introduction

Physical fitness can be considered the ability of the body systems to work effectively and efficiently, allowing our bodies to be healthy and to perform the activities of daily living with ease [1]. As a type of physical fitness, health-related physical fitness is a multi-component construct that includes cardiorespiratory endurance (aerobic power), musculoskeletal fitness, flexibility, balance, and speed of movement. According to the 2018 Physical Activity Guidelines Advisory Committee Scientific Report, physical fitness may serve as an intermediate variable along the pathway between physical activity and health outcomes and is associated with reduced all-cause mortality, cardiovascular disease mortality, and the risk of non-communicable diseases [2]. In addition to physical activity and weight status, physical fitness has been used as an appropriate public health outcome for children and adolescents [3]. Furthermore, fitness in childhood is considered an important indicator of current [4] and future [5] health, independent of physical activity [6].

As a physiologic characteristic, physical fitness is affected by both genetic and environmental factors. The available literature suggested that physical inactivity, increased sedentary time, and emerging obesity contributed to poor physical fitness [7]. Evidence also indicates that childhood cardiorespiratory fitness levels are declining in the United States [8], Canada [3,9], Sub-Saharan Africa [10], and worldwide [11]. Dong et al. recently reported that, since 1985, physical fitness among Chinese children and adolescents aged 7–18 (including respiratory function, strength, explosive power, and cardiorespiratory endurance) reached a peak in 1995 and then declined. Those authors also concluded that children and adolescents with both stunting and thin status and those with overweight and obese status had worse physical fitness than those with normal weight; however, age-specific physical fitness levels were lacking [12]. Our team also conducted cross-sectional research and found that physical fitness among children and adolescents aged 7–18 in Xinjiang was improved with normal weight status [13]. It has also been reported that physical fitness levels among children in Xinjiang declined during 2000–2005 [14]. However, secular trends in physical fitness among children and adolescents in Xinjiang remain unclear.

The Xinjiang Uygur Autonomous Region is an economically underdeveloped region located in northwest China, 2768 km from Beijing, China’s capital. Xinjiang covers an area of 1.66 million square kilometers and has 24.86 million inhabitants. Xinjiang is a multi-ethnic province comprising 46 ethnic groups, with Uyghur as the main population [15]. Ethnic groups such as Uyghurs, Kazaks, and Mongolians differ in many respects from the Han ethnic group in terms of religion, culture, diet, physical fitness, and lifestyle [16,17,18,19].

Owing to genetic and environmental differences from Han ethnicity, the Chinese national average physical fitness level may not represent the true physical fitness level among children and adolescents in Xinjiang. In the present study, we aimed to describe age and sex differences in physical fitness levels among young people in Xinjiang during 1985–2014 using data from four successive national surveys. We sought to assess future interventions designed to improve fitness levels at the population level and to increase our understanding of the relationships between fitness and health.

## 2. Materials and Methods

### 2.1. Participants

Data for the current study were drawn from the Chinese National Survey on Students’ Constitution and Health (CNSSCH), which is a nationwide survey that has been conducted every 5 years since 1985 by six government departments of China, including the Ministry of Education, the General Administration of Sport of China, and the National Health Commission of the People’s Republic of China, among others. In each survey, a thorough medical examination is conducted before taking measurements to exclude physical deformities or mental diseases. Participants in the CNSSCH include children and adolescents aged 7–18 across all 31 provinces of China. Considering socio-economic development level and geographic location, representative cities were selected from every province. Then, multistage stratified cluster sampling is used to select schools that have been relatively stable since 1985 and classes. The CNSSCH has been approved by the Medical Research Ethics Committee of Peking University Health Science Center (IRB00001052-18002). Written informed consent was obtained before the survey and all participants’ names were digitally coded to protect personal information.

In the present study, the data of a total 49,357 children and adolescents in Xinjiang were extracted from the CNSSCH in 1985 (n = 14,548), 1995 (n = 7198), 2005 (n = 10,255), and 2014 (n = 17,356).

### 2.2. Procedure and Measurement

All research technicians in this study, recruited from among middle and high school physical education (PE) teachers, were trained using a standardized testing manual illustrating all test guidelines, procedures, and protocols. Measurement of all items followed a standardized procedure and was performed by trained technicians, according to CNSSCH guidelines [20]. All tests were completed in one day via an assembly line to decrease testing error.

Portable stadiometers were used to measure the height (cm) to the nearest 0.1 cm, and standardized scales were used to measure weight (kg) to the nearest 0.1 kg, with participants wearing light clothing and no shoes. Body mass index (BMI; kg/m^2^) was calculated as weight (kg) divided by height (m) squared.

Six items were measured to assess physical fitness, including forced vital capacity (FVC), standing long jump, sit-and-reach, body muscle strength, 50-m dash, and endurance running. FVC was measured to reflect respiratory function using a rotary spirometer in 1985; a needle spirometer and electronic spirometer were added in 1995. Although different spirometry methods have been used owing to the updating of instruments, the principles underlying the methods and instruments are the same. Body muscle strength includes lower limb strength and upper body strength. We used a standing long jump to assess lower limb strength and oblique body pull-ups for boys aged 7–12, pull-ups for boys aged 13–18, and 1-min sit-ups for all girls aged 7–18, to assess upper body muscle strength. The cardiopulmonary function was assessed with long-distance running (eight 50-m shuttle runs for participants aged 7–12, 1000-m endurance running for boys aged 13–18, and 800-m endurance running for girls aged 13–18). The 50-m dash was used to assess speed of movement.

### 2.3. Growth and Nutritional Status and Physical Fitness Indicator (PFI)

BMI-for-age Z-scores and height-for-age Z-scores were calculated by the sex and age group, with reference to the 2007 World Health Organization BMI standards. Stunting was defined as height-for-age Z-score < −2 to represent growth status. Nutritional status was classified as underweight (< −2 for BMI Z-score), normal (≥ −2 and ≤ 1 for BMI Z-score and ≥ −2 for height Z-score), overweight (> 1 and ≤ 2 for BMI Z-score), or obese (> 2 for BMI Z-score).

To overcome difficulties during comparisons caused by the different units and sexes, sex-specific and age-specific standardized values for each core item were calculated using the 1985 survey year dataset as the reference population. The PFI was then obtained by combining Z-scores of the six core items, to assess physical fitness at both individual and population levels [12,21]. It should be noted that Z-scores for the 50-m dash and endurance run were reversed because lower times reflect better performance in these two tests.

### 2.4. Statistical Analyses

Missing data or extreme values for height, weight, and physical fitness items were excluded on the basis of the logical check boundary provided by the CNSSCH association [20]. Demographic information and values for the six core items of physical fitness were summarized as means and standard deviations. The trends in PFI and the six core items of physical fitness with different ages and growth and nutrition statuses were evaluated between 1985 and 2014. After the Kolmogorov–Smirnov test of normality, the comparisons of the PFI of 1985, 1995, 2005, 2014 were conducted by nonparametric Kruskal–Wallis test for the four age groups within each sex, followed by pairwise comparison. The relationship between BMI and PFI for the survey was investigated using a nonlinear regression model, as follows: PFI = aBMI^2^ + bBMI + c; where a, b, c are constants. PFI was used as the dependent variable, and BMI was considered the independent variable. The level of statistical significance was set at 0.05. All analyses were conducted using IBM SPSS version 23.0 (IBM Corp., Armonk, NY, USA) and GraphPad Prism 8.0.2(GraphPad Software, Inc., CA, USA).

## 3. Results

The data of a total of 49,357 children and adolescents in Xinjiang, aged 7–18, for 1985, 1995, 2005, and 2014, were extracted from the CNSSCH for the current study. Table 1 shows that the proportions of the study population by sex and age groups were consistent across the four survey years. Continuous increases in average height, weight, and BMI were observed from 1985 to 2014. In terms of nutritional status, the prevalence of stunting and underweight declined from 10.3% and 1.7% to 6.5% and 0.4%, respectively. At the same time, the prevalence of overweight and obesity increased by 92.5% and 98.7% in 2014, as compared with 1985. The prevalence of normal weight increased from 87.5% in 1985 to 89.4% in 1995 and then decreased to 75.2%, lower than the prevalence in 1985.

Comprising the six core physical fitness items, the PFI increased to 2 in 1995 and then fell sharply to −2.8 in 2005 and continued to decrease to −3.8 in 2014, taking the 1985 dataset as reference. In terms of specific items, the trend in FVC, standing long jump, sit-and-reach, 50-m dash, and body muscle strength were consistent with the PFI except for FVC and pull-ups in boys aged 13–18, which increased from 2005 to 2014 but remained worse than in 1985. The performance of endurance running remained relatively stable during 1985–1995 but was worse in 2005 and 2014 (Table 2).

Table 3 shows the trend in the PFI of children and adolescents in Xinjiang by age group and sex during 1985–2014. The PFI of all age groups reached a peak in 1995, followed in descending order by 1985, 2005, and 2014, except for the PFI in boys aged 13–15 and 16–18 in 2014, which was slightly increased. From 1985 to 1995, the largest increment occurred in boys aged 13–15 (by 2.8) and girls aged 16–18 (by 2.5). From 1995 to 2005, the largest decline occurred in adolescents aged 10–12, by −5.5 in boys and by −5.3 in girls. The trend seemed better for adolescents aged 13–18 than for children aged 7–12. All changes among the different age groups and years were significant (*P* < 0.001).

**Figure 1:**
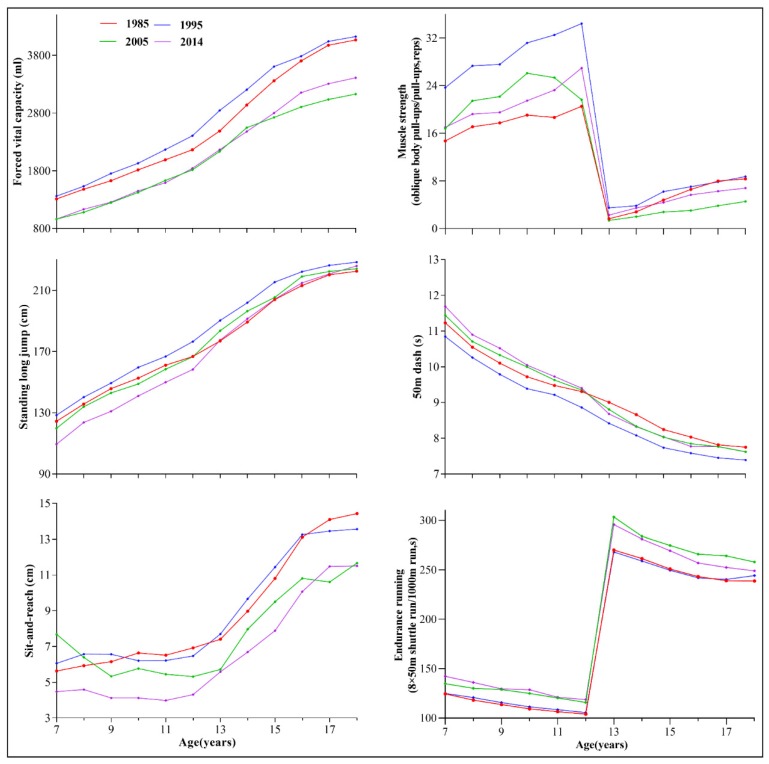
Trends in the six core items of physical fitness among boys aged 7–18 in Xinjiang, from 1985 to 2014.

**Figure 2:**
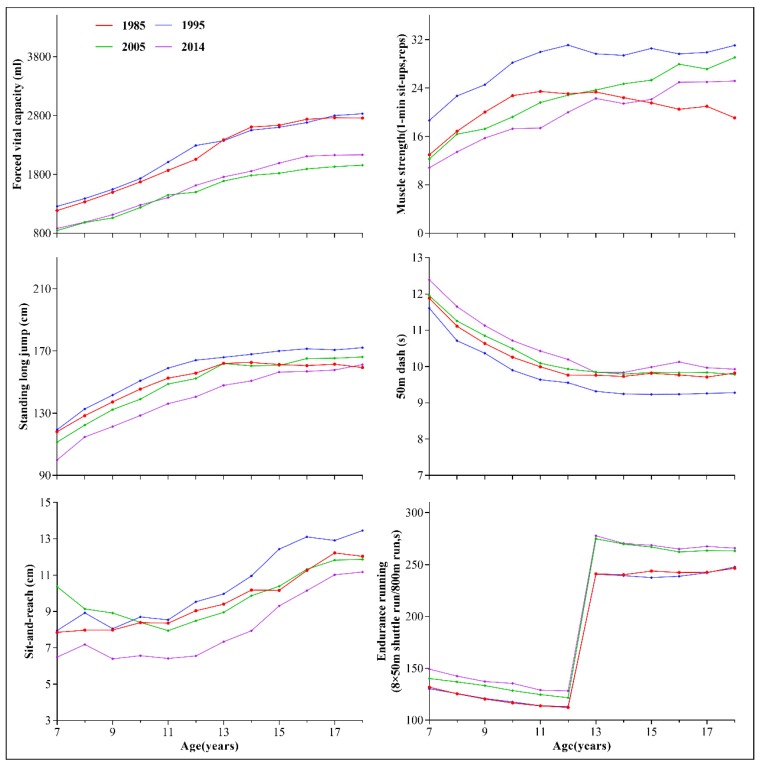
Trends in the six core items of physical fitness among girls aged 7–18 in Xinjiang, from 1985 to 2014.

Figure 1 and Figure 2 shows the trends in the six core items of physical fitness among children and adolescents in Xinjiang aged 7–18, from 1985 to 2014. The components of fitness increased over time from 1985 to 1995 in all age groups (age 7–18) except for sit-and-reach and endurance running, which remained relatively stable. However, during 1995–2005, performance declined for all age groups, especially for FVC. Although most elements of physical fitness declined from 2005 to 2014, it should be noted that we observed an increment in FVC among adolescents aged 13–18 and muscle strength in boys aged 13–18.

**Figure 3:**
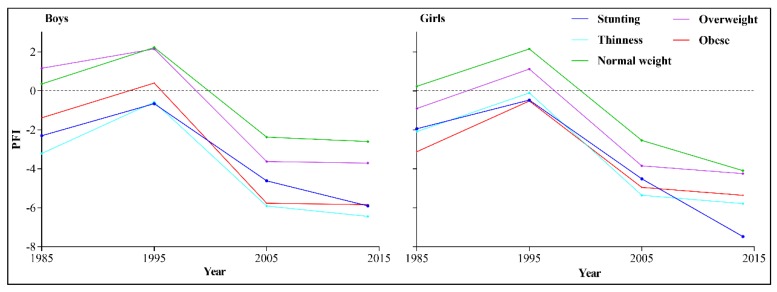
Trend in the physical fitness indicator (PFI) of children and adolescents in Xinjiang, according to nutritional status, 1985 to 2014.

Figure 3 shows the trend in PFI among children and adolescents in Xinjiang, according to different nutritional status, from 1985 to 2014. Compared with 1985, the PFI of all children and adolescents reached its peak in 1995 and then experienced a sharp decline in 2005, followed by a slight decline in 2014. The PFI of children and adolescents with normal weight in all years was higher than in those with stunting, underweight, overweight, and obesity.

**Figure 4:**
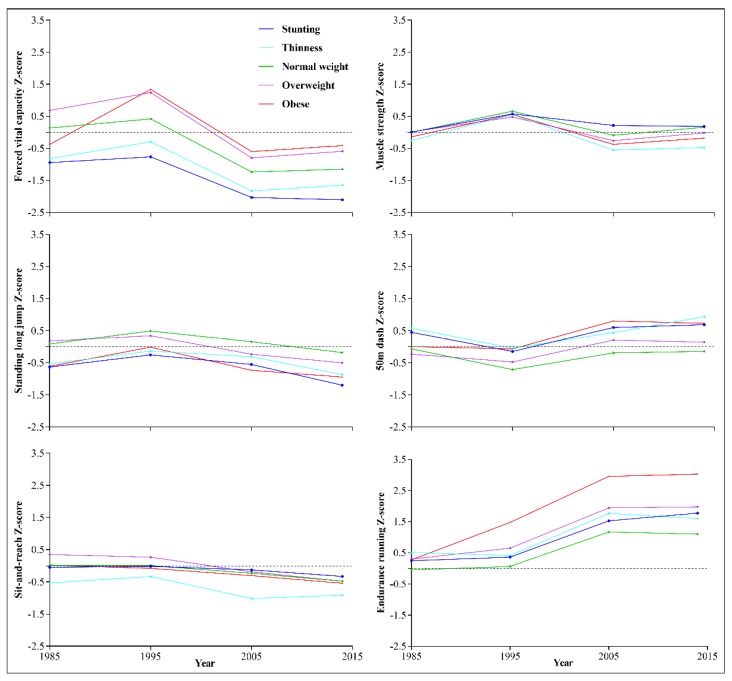
Trends in the six core items of physical fitness among boys in Xinjiang with different nutritional status, 1985 to 2014.

**Figure 5:**
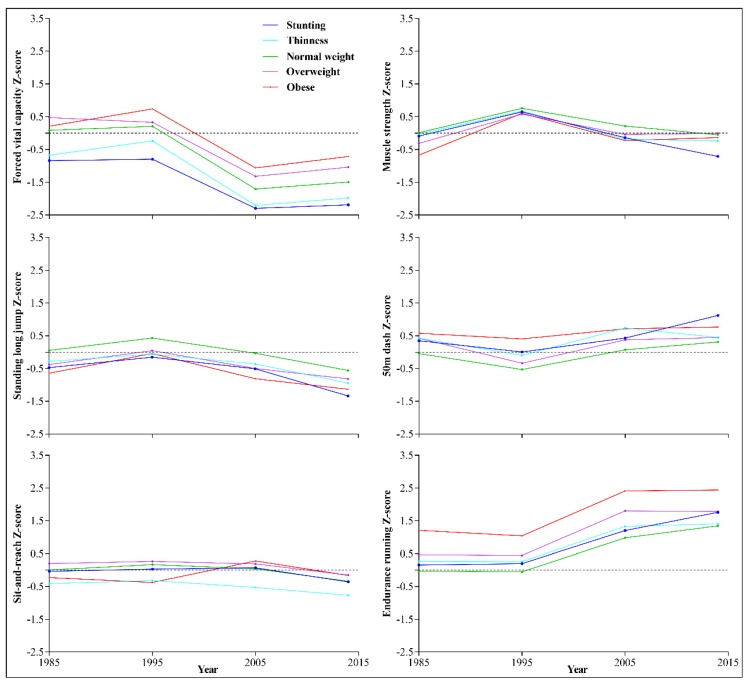
Trends in the six core items of physical fitness among girls in Xinjiang with different nutritional status, 1985 to 2014.

Trends in the six core items of physical fitness among children and adolescents in Xinjiang during 1985-2014, according to nutritional status, are shown in Figure 4 and Figure 5. Overall, except for FVC and sit-and-reach, students with normal weight status had the best results, whereas those with obesity showed the lowest results for each measure of physical fitness during 1985–2014. Among the six core items, FVC and endurance running showed the greatest changes during 1995–2005, especially endurance running for the group with obesity. Students with obese status had the highest FVC and sit-and-reach results, whereas students with stunting had the lowest FVC and those with underweight had the lowest sit-and-reach results.

**Figure 6:**
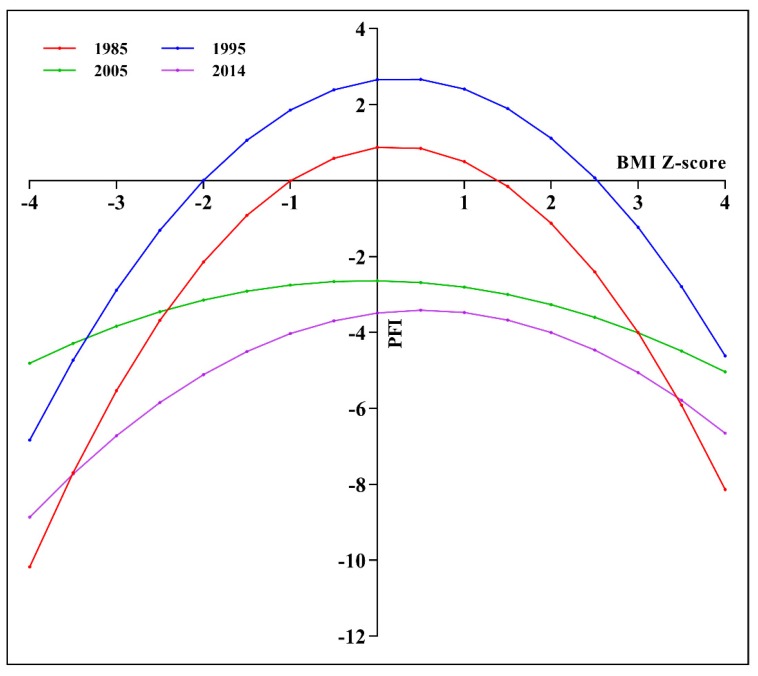
Relationship between body mass index (BMI) Z-score and PFI Z-score for children and adolescents in Xinjiang, 1985 to 2014.

We also estimated the relationship between BMI Z-score and PFI Z-score for children and adolescents aged 7–18 in Xinjiang from 1985 to 2014 and revealed inverted U-shaped curves. This result indicates that higher or lower BMI had a negative effect on the PFI of children and adolescents (Figure 6).

## 4. Discussion

We assessed secular trends in the PFI of children and adolescents aged 7–18 in Xinjiang from 1985 to 2014 using the data of 49,357 individuals extracted from four rounds of the CNSSCH. The current study’s findings suggest that the PFI of this group of children and adolescents reached its peak in 1995, with the largest increment in boys aged 13–15 and girls aged 16–18. We also observed a sharp decline (by 290%) in the PFI of these children and adolescents in 2014 as compared with 1995, with the largest decline occurring in both boys and girls aged 10–12. Consistent with the trend in PFI, during 1985–2014, the prevalence of normal weight also peaked in 1995. Children and adolescents with both overnutrition and undernutrition had poorer physical fitness than those with normal weight and height. The inverted U-shaped curves obtained for the relationship between BMI Z-score and PFI Z-score indicated that PFI can improve with normal weight status.

Our study’s findings suggest that, from 1985 to 2014, the PFI of children and adolescents aged 7–12 in Xinjiang showed a greater decline than in those aged 13–18. This might be explained by the inclusion of PE in both senior high school entrance examinations (students participate at age 15) and college entrance examinations (students participate at age 18); however, primary school entrance examinations were not included (students participate at age 12). This also reveals the shortcomings of China’s PE classes and exam-oriented PE curriculum. Recently, the Chinese healthy PE curriculum model [22,23], developed by Professor Ji was introduced, aiming to improve students’ physical fitness by helping students to enjoy PE and engage in at least one sport as a hobby. At present, this model has been promoted throughout the country and a large number of Chinese PE teachers have been trained in the use of the model [24,25,26]. More effective teaching models must be developed, to promote physical fitness in Chinese students.

The present study showed that both malnutrition and overnutrition have adverse effects on physical fitness, which is consistent with previous studies [27,28]. The prevalence of stunting and underweight in 1985 was 10.3% and 1.7%, respectively, and the prevalence of overweight and obesity combined was 0.9%; the former was likely the cause of the decline in physical fitness. Whereas, in 2014, the prevalence of stunting and underweight declined to 6.5% and 0.4%, respectively, and the prevalence of overweight and obesity combined increased to 18.3%, and the latter may have driven the decline in physical fitness. Widespread obesity, which is caused by increased physical inactivity and sedentary time, has become a public health issue all over the world, including in China [29,30,31]. Improving physical fitness is undoubtedly the best choice for increasing physical activity and reducing sedentary time among children and adolescents in Xinjiang [32,33].

Given the low rates of participation in physical activity globally, the decline in physical fitness in the current study is partly affected by physical inactivity in children and adolescents, which has profound implications [34]. Strong evidence demonstrates that higher levels of physical activity are associated with multiple beneficial health outcomes, including cardiorespiratory and muscular fitness, bone health, and the maintenance of healthy weight in children and adolescents [35]. The 2018 Physical Activity Guidelines Advisory Committee Scientific Report overwhelmingly demonstrates that physical activity is a “best buy” for public health [2]. The World Health Organization has issued the global health recommendation that children and adolescents should engage in moderate to vigorous physical activity for at least 60 min a day [36]. Unfortunately, only 30% of Chinese children and adolescents meet this recommendation for daily physical activity [37]. As an underdeveloped province in China, the sports facilities in schools and communities of Xinjiang are relatively lacking [38]. According to the results of the fifth national survey of sports venues in China during 2004 [39], two-thirds of primary schools in Xinjiang lacked regular sports venues, especially in rural areas. In addition, parents’ education level is low, and the holding rate of health literacy was only 2.1% for Xinjiang residents in 2010, which is much lower than the national average [40]. Sedentary behavior has been associated with a wide range of negative health indicators, including obesity, poor cardiometabolic health, and poor psychosocial health [41]. Studies of Chinese adolescent populations have shown a rising trend in sedentary behavior [42] and that 37% of Chinese school-aged children do not adhere to the daily screen-viewing recommendations of 2 h or less [43]. Thirty years of economic development in China have brought about a behavioral shift from traditionally active lifestyles to more industrialized and sedentary lifestyles. According to the Internet development report of Xinjiang Uygur Autonomous Region in 2016, Internet penetration reached 54.9%, which is higher than the national average [44]. Therefore, current efforts should focus on effectively improving physical activity and reducing sedentary behavior as well as improving nutritional status. Improving physical fitness among children and adolescents requires joint efforts by schools, families, and communities [34]. In addition to the home, school is another place where students spend most of their time; thus, school may be a priority target for improving exercise and promoting physical fitness among students [45].

With a heavy schoolwork burden and college entrance examination pressure, the sedentary time has increased rapidly in recent years, which may be the cause of physical decline among Chinese children and adolescents, especially during 1995–2005. This continuous decline in physical fitness among children and adolescents has attracted the attention of the Chinese government, which has established many policies to promote physical health, such as the “Healthy China 2030” program [46], seeking to make time for more PE classes and the practice of an hour of exercise per day [47] as well as increasing investment in school sports facilities. The gross domestic product of Xinjiang was ranked fifth lowest in China in 2014, and the urbanization rate was 46.07%, which is also a relatively low level [48]. The published literature suggests that attention should be focused on undernutrition rather than overnutrition to promote physical fitness in less-developed regions of China [49,50], whereas in developed regions, issues of overnutrition should be on the agenda. Therefore, to improve the physical fitness levels of children and adolescents in Xinjiang, specific policies should be set according to the actual nutritional and socio-economic development status. Nutrition subsidies and nutrition projects should be carried out in southern Xinjiang and rural areas, where undernutrition remains a serious problem [51]. In contrast, consumption should be limited by raising taxes on energy- and sugar-rich foods and drinks in provincial capital cities and urban areas, where the prevalence of overweight and obesity are higher [52]. In addition, actions should be taken to develop a healthy lifestyle and regular sleep habits and to promote physical fitness.

An advantage of having fitness measures from the four surveys is the consistency among measurement protocols. However, there are some limitations to the current study. First, we only evaluated the impact of nutritional status on physical fitness, whereas other factors, such as diet, physical exercise, and family environment were not included; these may be important factors affecting physical fitness among children and adolescents in Xinjiang. Second, a standardized and accepted method is lacking for assessing physical fitness at present. The PFI was assessed using the combination of performance results for FVC, standing long jump, sit-and-reach, body muscle strength, 50-m dash, and endurance running in the present study. The physical fitness level of children and adolescents in Xinjiang may not be comprehensive and accurately evaluated using PFI; however, this indicator can still represent physical fitness levels to some extent^12^.

## 5. Conclusions

This study provides an update regarding the physical fitness of children and adolescents in Xinjiang, China. The results demonstrate that physical fitness levels reached their peak in 1995, followed by continuous declines in 2005 and 2014. The lack of progress in physical fitness suggests that efforts to improve fitness and behaviors of healthy, active living among children and adolescents in Xinjiang have been insufficient; thus, ongoing enhanced efforts are required. Students with overnutrition and children age 7–12 require special attention. Effective new teaching models are also needed, such as the Chinese healthy PE curriculum model, rather than focusing solely on PE examinations.

## Figures and Tables

**Table 1:** Sample distribution and nutrition status among children and adolescents aged 7–18 in Xinjiang, 1985 to 2014.

Category	1985	1995	2005	2014
Sample size	14,548	7198	10,255	17,356
Sex				
Boys (%)	7261(49.9)	3600(50.0)	5138(50.1)	8671(50.0)
Girls (%)	7287(50.1)	3598(50.0)	5117(49.9)	8685(50.0)
Age groups (years)				
7-9yr (%)	3628(24.9)	1800(25.0)	2441(23.8)	4378(25.2)
10-12yr (%)	3637(25.0)	1800(25.0)	2625(25.6)	4263(24.6)
13-15yr (%)	3650(25.1)	1798(25.0)	2603(25.4)	4306(24.8)
16-18yr (%)	3633(25.0)	1800(25.0)	2586(25.2)	4409(25.4)
Nutritional status				
Height (cm, SD)	146.19(16.82)	148.35(16.69)	149.91(16.94)	149.17(17.12)
Weight (kg, SD)	37.22(12.88)	38.99(13.03)	41.88(14.50)	43.27(14.80)
BMI (kg/m^2^, SD)	16.76(2.56)	17.10(2.63)	18.01(3.20)	18.79(3.20)
Stunting (%)	1504(10.3)	456(6.3)	503(4.9)	1136(6.5)
Underweight (%)	250(1.7)	121(1.7)	115(1.1)	78(0.4)
Normal weight (%)	12731(87.5)	6438(89.4)	8436(82.3)	13051(75.2)
Overweight (%)	114(0.8)	177(2.5)	673(6.6)	1835(10.6)
Obese (%)	12(0.1)	28(0.4)	564(5.5)	1343(7.7)

Abbreviation: SD, standard deviation.

**Table 2:** Six core items of physical fitness among children and adolescents aged 7–18 in Xinjiang, from 1985 to 2014.

Physical Fitness Status	1985	1995	2005	2014
Physical fitness indicator (SD)	0	2(3.7)	−2.8(4.1)	−3.8(4.8)
Forced vital capacity (ml, SD)	2350.6(918.4)	2450.7(944.1)	1797(816.5)	1868.3(866.7)
Standing long jump (cm, SD)	163.2(33.1)	170.5(34.1)	163.5(35.6)	155(39.9)
Sit-and-reach (cm, SD)	9.2(5.5)	9.7(5.5)	8.7(6.2)	7.3(6.1)
50 m dash (s)	9.7(1.3)	9.3(1.3)	9.7(1.4)	9.9(1.6)
Body muscle strength				
Number of oblique body pull-ups (boys aged 7–12, SD)	18.0(11.8)	29.4(14.7)	22.3(17.2)	21.2(14.1)
Number of pull-ups (boys aged 13–18, SD)	5.3(4.6)	6.2(4.3)	2.9(3.7)	4.8(4.3)
Number of 1-min sit-ups (girls aged 7–18, SD)	20.6(10.3)	28.0(9.8)	22.4(10.5)	19.7(10.8)
Endurance running (s)				
8×50 m shuttle run (students aged 7–12, SD)	116.4(11.6)	117.3(12.2)	128.0(14.2)	133.3(17.4)
1000 m run (boys aged 13–18, SD)	250.6(25.3)	250.5(26.7)	274.8(34.9)	267.1(41.3)
800 m run (girls aged 13–18, SD)	242.8(24.8)	241.1(24.2)	266.8(33.4)	269.3(37.9)

**Table 3:** Physical fitness indicators of children and adolescents in Xinjiang for different age groups, 1985 to 2014.

Age (yr)	1985		1995		2005		2014		H	*P*
	N	Mean (SD)	N	Mean (SD)	N	Mean (SD)	N	Mean (SD)		
Boys										
7-9yr	1817	0	900	1.7(3.6)	1212	−2.9(4.4)	2192	−5.0(4.2)	1872.74	<0.001
10-12yr	1819	0	900	2.3(4.2)	1316	−3.2(5.0)	2153	−4.6(5.3)	1502.82	<0.001
13-15yr	1822	0	900	2.8(4.3)	1306	−1.9(4.1)	2140	−1.5(4.9)	699.78	<0.001
16-18yr	1803	0	900	1.1(3.3)	1304	−3.5(3.7)	2186	−2.1(4.3)	917.17	<0.001
Girls										
7-9yr	1811	0	900	1.5(3.5)	1229	−2.9(3.9)	2186	−5.3(4.4)	1940.52	<0.001
10-12yr	1818	0	900	1.9(3.7)	1309	−3.4(4.0)	2110	−5.8(4.9)	2061.45	<0.001
13-15yr	1828	0	898	1.9(3.1)	1297	−3.1(3.7)	2166	−4.0(4.4)	1665.85	<0.001
16-18yr	1830	0	900	2.5(3.2)	1282	−1.8(3.6)	2223	−2.5(4.0)	1132.59	<0.001

Note: all the differences were significant (*P* < 0.001) in pairwise comparison of 1985, 1995, 2005, and 2014 for all age groups within each sex.
